# Effect of environmental history on the habitat-forming kelp *Macrocystis pyrifera* responses to ocean acidification and warming: a physiological and molecular approach

**DOI:** 10.1038/s41598-021-82094-7

**Published:** 2021-01-28

**Authors:** Pamela A. Fernández, Jorge M. Navarro, Carolina Camus, Rodrigo Torres, Alejandro H. Buschmann

**Affiliations:** 1 Centro i~mar and CeBiB, Universidad de Los Lagos, Camino Chinquihue km 6, Puerto Montt, Chile; 2 Instituto de Ciencias Marinas y Limnológicas and Centro Fondap de Investigación de Ecosistemas Marinos de Altas Latitudes (IDEAL), Universidad Austral de Chile, Valdivia, Chile; 3 Centro de Investigación en Ecosistemas de la Patagonia (CIEP), José de Moraleda 16, Coyhaique, Chile

**Keywords:** Molecular biology, Physiology

## Abstract

The capacity of marine organisms to adapt and/or acclimate to climate change might differ among distinct populations, depending on their local environmental history and phenotypic plasticity. Kelp forests create some of the most productive habitats in the world, but globally, many populations have been negatively impacted by multiple anthropogenic stressors. Here, we compare the physiological and molecular responses to ocean acidification (OA) and warming (OW) of two populations of the giant kelp *Macrocystis pyrifera* from distinct upwelling conditions (weak vs strong). Using laboratory mesocosm experiments, we found that juvenile *Macrocystis* sporophyte responses to OW and OA did not differ among populations: elevated temperature reduced growth while OA had no effect on growth and photosynthesis. However, we observed higher growth rates and NO_3_^−^ assimilation, and enhanced expression of metabolic-genes involved in the NO_3_^−^ and CO_2_ assimilation in individuals from the strong upwelling site. Our results suggest that despite no inter-population differences in response to OA and OW, intrinsic differences among populations might be related to their natural variability in CO_2_, NO_3_^−^ and seawater temperatures driven by coastal upwelling. Further work including additional populations and fluctuating climate change conditions rather than static values are needed to precisely determine how natural variability in environmental conditions might influence a species’ response to climate change.

## Introduction

Anthropogenic climate change, such as global warming and ocean acidification (OA) are altering the structure and functioning of terrestrial and marine ecosystems, causing shifts in the distribution and relative abundance of species^1–4^. In marine environments, ocean warming (OW), OA and associated changes in the seawater carbonate chemistry (i.e., increases in [HCO_3_^−^] and [H^+^] but decreases in the carbonate saturation state (Ω) are expected to have direct and indirect physiological and ecological impacts on calcifying and non-calcifying organisms, affecting metabolic processes such as growth, calcification and photosynthesis^5–8^. In non-calcifying macroalgae, the magnitude and direction of these responses can be influenced by the interaction with local factors that play an important role in their performance, such as nutrients and light availability^9–14^. Local exposure to different regimes of environmental drivers (e.g., temperature, light, nutrients and pCO_2_/pH) can drive divergent selection among populations^15^, and hence might influence macroalgal responses to predicted environmental changes associated with climate change.

Coastal ecosystems are strongly influenced by inputs of freshwater, upwelling events and eutrophication, all of them affecting the carbonate system and biogeochemical processes^16–19^. For example, upwelling events cause a temporary reduction in pH (< 8.0) and aragonite saturation states (Ω_arag_), usually reaching values similar to those projected by the next century^16,20–23^. Moreover, in upwelling systems, pCO_2_ concentrations can result higher (> 600 µatm) than current ambient concentrations (400 µatm)^22,24,25^. As a result, species (or populations) inhabiting these highly fluctuating environments may have evolved phenotypic differences as well as physiological and genetic mechanisms that provide advantages in fitness in their local environment^26^, which can be of great importance for organism resilience to ongoing climate change^6,11,27^. For example, divergent population responses to OA have been observed among calcifying invertebrates and corals that experience different pCO_2_/pH regimes^24,28–30^. These responses have been mostly driven by their natural environmental variability (e.g., weak upwelling vs strong upwelling) that influences their tolerance and responses to climate change^27^. The coastal area of Central Chile (~ 33°S) is characterized by seasonal, wind-driven upwelling that brings cold nutrient-rich waters to the surface in austral spring and early summer seasons^31–37^. The process is intensified around capes, and modified by coastline orientation, generating variation in SSTs over scales of a few to 10 s of kilometers^38,39^. Within the study region, Punta Curaumilla (~ 33°06′S) is one of the main upwelling centres of the Chilean coast^36,37,40^ in which upwelled waters are characterized by cold (< 14 °C), low dissolved oxygen, high salinity (> 34 psu) and nutrient-rich conditions (phosphate > 2.0 µM; nitrate > 15 µM; silicate > 10 µM)^33^. In contrast, some areas (e.g., El Tabo located at 33°27′S) are weakly or indirectly influenced by these events^31^ with organisms experiencing less environmental variability in temperature (~ 16 °C) and nitrate concentrations (< 5 µM) during the spring–summer upwelling season^32,34,38,41^. Moreover, previous studies have shown that the abundance of algal functional groups like kelps can be modified by natural variation in nutrient concentrations driven by upwelling, with upwelling areas showing greater macroalgal biomass than those where these events are less frequent^34,41–43^. However, there is limited understanding of how natural variability in temperature (cooling) and pCO_2_/pH regimes driven by coastal upwelling might influence macroalgal population responses to OA or other environmental changes driven by climate change (e.g., OW).

Kelps, large brown macroalgae of the order Laminariales, are widely distributed across temperate and subpolar regions, creating some of the most diverse and productive habitats in the world^44–49^. Therefore, declines in their abundances can have severe consequences for the entire associated ecosystem, causing a shift toward a less diverse and productive ecosystem^3,12,50^. Across their wide distribution, these species are exposed to a variety of environmental conditions and are capable of acclimating to a wide range of abiotic and biotic factors^51,52^. However, they are particularly susceptible to warm water temperatures and low nutrient availability (i.e., inorganic nitrogen)^53^, and to marine heat wave events when located close to their equatorward margins^54^. Although kelp ecosystems have been severely affected by OW and marine heat wave events, region-specific responses have also been detected, with some kelp populations increasing or remaining stable over the past 50 years^55^. This suggests that kelp’s responses to global climate changes can be influenced by local driver interactions but also by their environmental history as previously shown in key reef-building taxa (corals and coralline algae)^27^.

Kelp species seem to have evolved different physiological capabilities to deal with fluctuating environmental conditions, making it difficult to generalize how these organisms can respond to climate change^56,57^. For example, local adaptation to changes in nitrogen variability^58,59^, temperature^60–64^ and pH^63^ have been observed across isolated populations of kelps (i.e., *Macrocystis pyrifera, Laminaria longicruris*, *Undaria pinnatifida).* In the specific case of the giant kelp *M. pyrifera* (thereafter, *Macrocystis*), which is widely distributed throughout cold temperate waters of both hemispheres^49,65^, a wide physiological plasticity and genetic diversity has been described across its geographic range^66,67^. In Chile, populations of *Macrocystis* are exposed to natural variability in pCO_2_/pH, nutrients, and temperature driven by upwelling and El Niño events (warming conditions), both simulating near-future predicted scenarios (i.e., OA and OW). It has been recently shown that microscopic life stages coming from individuals that are experiencing strong coastal upwelling are less vulnerable to OA conditions^63^. However, the physiological and molecular mechanisms promoting these differences are still poorly understood in this species. Moreover, as microscopic and macroscopic life stages of *Macrocystis* have different physiological requirements, differential responses to environmental changes driven by climate change might be expected across its life history stages (e.g., gametophytes, juveniles and adults)^68–71^.

In the present work we investigated and compared the physiological (growth, photosynthesis, enzyme activities), biochemical (total tissue nitrogen and carbon content) and molecular responses (expression of specialized genes involved in carbon and nitrogen metabolism) of juvenile *Macrocystis* sporophytes to OW and OA from populations naturally exposed to different environmental variability (weak upwelling vs strong upwelling). The aim of the present study was to determine whether *Macrocystis* responses to OA and OW are influenced by their environmental history. We hypothesized that (1) juvenile sporophytes from populations exposed to highly fluctuating environments (strong more variable upwelling) will be more tolerant to increased pCO_2_ and high temperature than those from less fluctuating environments (weak less variable upwelling; hereafter “weak upwelling”), showing, e.g., higher growth and photosynthetic rates. (2) The expression of metabolic-genes related to nitrogen (nitrate reductase, NR) and carbon (carbonic anhydrase, CA) metabolism will show differential expression among populations, depending on their natural variability in NO_3_^−^ and pCO_2_ concentrations. To do this, we grew *Macrocystis* early life stages from two populations (Las Docas: hereafter “strong upwelling site”; El Tabo: hereafter “weak upwelling site”) under the same experimental conditions (acclimation period). After four months, juveniles were incubated under two CO_2_/pH levels (ambient and future OA scenario) and three temperature treatments (12 °C, 16 °C and 20 °C that simulated winter, summer and OW SSTs, respectively) for 21 days.

## Results

### Physiological traits and biochemical composition

**Figure 1:**
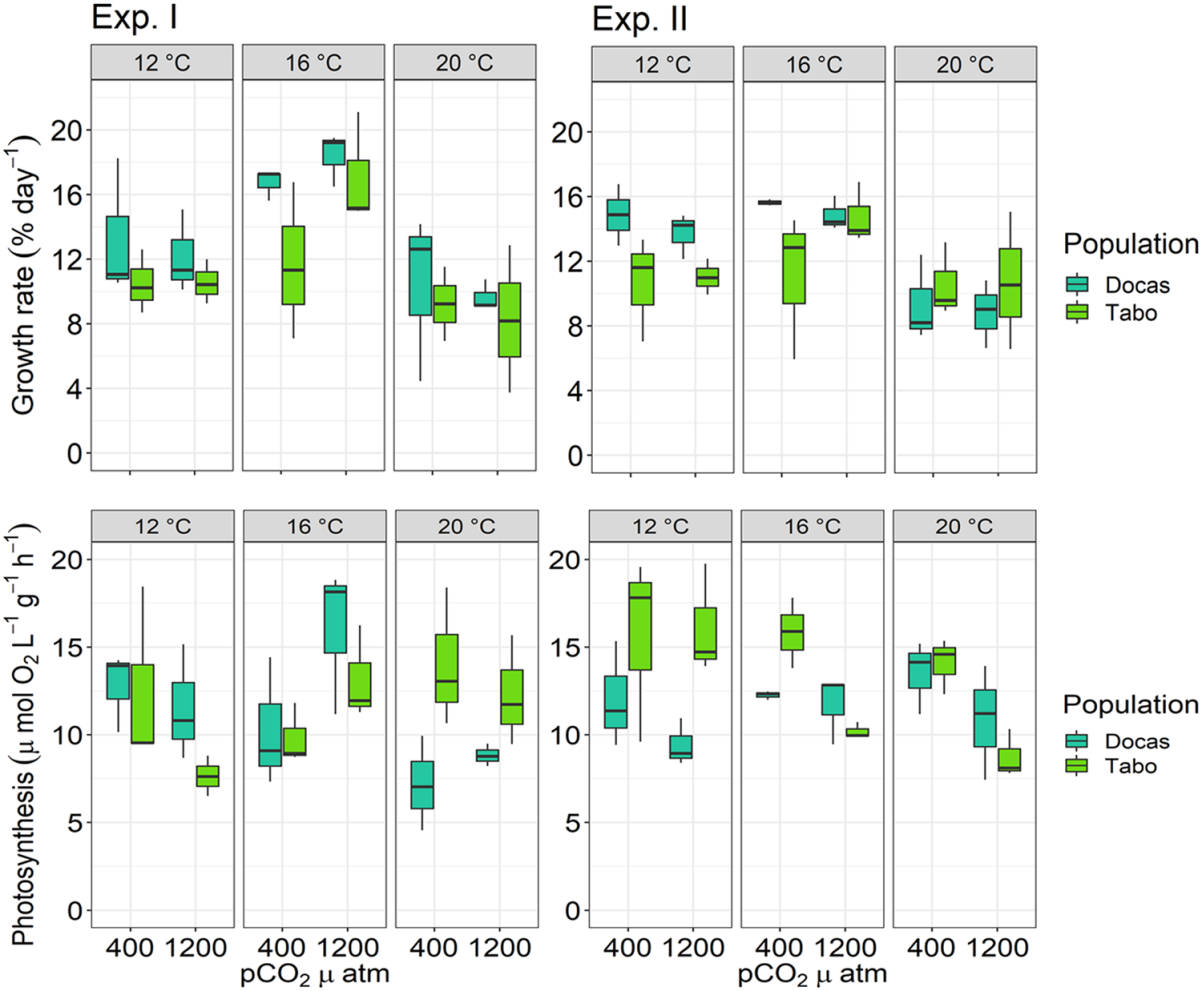
Relative growth rates (% day^−1^) and photosynthesis of juvenile *Macrocystis* sporophytes from Las Docas and El Tabo, incubated for three weeks (Experimental trial I and II) under three temperature (12, 16 and 20 °C) and two pCO_2_ treatments (~ 400 and 1200 µatm). Box-plots showing median size (line within box), 25th and 75th percentiles (ends of box), and minimum and maximum distribution of the data (whisker).

Relative growth rates (RGR, % day^−1^) of juvenile *Macrocystis* sporophytes were affected by temperature (*p* = 1.505e^−07^) and location (*p* = 0.014) but not by pCO_2_/pH (all statistical results are summarized in Supplementary Table S3, Fig. 1). No difference in growth responses was observed across experimental trials (Table 1). RGRs of juvenile sporophytes from both populations were higher at 16 °C (16.17 ± 0.85% day^−1^ and 15.38 ± 1.78% day^−1^, strong and weak upwelling sites, respectively) followed by 12 °C (Tukey test, *p* =  < 0.001), but decreased by 30% at 20 °C (9.87 ± 3.75% day^−1^ and 9.48 ± 4.16% day^−1^; strong and weak upwelling sites, respectively, Tukey test, *p* = 0.005). Moreover, RGRs varied among populations, with individuals from the strong upwelling site showing higher RGRs than those from the weak upwelling site across the experimental treatment combinations (Tukey test, *p* = 0.005; Fig. 1).

**Table 1:** Physiological, biochemical and molecular responses of *Macrocystis pyrifer*a juveniles to changes in temperature and pCO_2_ across locations and trials.

Traits	Temperature	pCO_2_	Location	Trial	Statistical interactions
Growth rate	< 0.0001 ↓	NS	0.014	NS	–
Photosynthetic rate	NS	NS	NS	NS	–
Alpha (*α*)	NS	NS	NS	NS	–
ETR_max_	NS	NS	NS	0.033	–
Ek	NS	NS	NS	NS	–
Fv/Fm	0.031 ↓	NS	NS	0.047	–
NR activity	NS	NS	0.01	0.0001	–
CA activity	0.001	NS	0.008	NS	–
Chl *a*	< 0.001	NS	NS	NS	Temp × Trial Location × Trial
Chl *c*	NS	NS	0.002	0.001	Temp × Trial Location × Trial Temp × Location × Trial
Fucoxanthin	0.001 ↑	NS	NS	0.041	Temp × Location Temp × Trial Location × Trial Temp × Location × Trial
Tissue *N* content	< 0.0001 ↓	NS	0.031	< 0.0001	-
Tissue *C* content	< 0.001 ↑	NS	< 0.001	< 0.001	pCO_2_ × Temp × Location
*C*/*N* ratio	< 0.0001	NS	NS	NS	–
NR gene expression	< 0.0001 ↓	0.008 ↑	0.0100	–	Temp × Location
CA gene expression	< 0.0001 ↓	NS	< 0.0001	–	pCO_2_ × Location Temp × Location pCO_2_ × Temp × Location
SP gene expression	0.0002 ↓	NS	NS	–	Temp × Location

NS represents no significant effect, *p* values < 0.05 represent significant effect, “↑” represents increase and “↓” represents decrease.

**Table 2:** Seawater carbonate chemistry parameters (i.e. pH_T_, A_T_, pCO_2_, HCO_3_^−^, CO_3_^2−^, CO_2_), salinity and nutrient concentrations (i.e. NO_3_^−^, Si, NH_4_^+^, PO_4_^3−^) at the start of each experimental trial (I–II).

Parameter	Experimental trial I						Experimental trial II					
	Ambient pCO_2_			OA scenario			Ambient pCO_2_			OA scenario		
	12 °C	16 °C	20 °C	12 °C	16 °C	20 °C	12 °C	16 °C	20 °C	12 °C	16 °C	20 °C
pH_T_	7.72 ± 0.003	7.73 ± 0.010	7.78 ± 0.006	7.49 ± 0.04	7.46 ± 0.007	7.54 ± 0.001	7.80 ± 0.0007	7.77 ± 0.03	7.88 ± 0.014	7.46 ± 0.008	7.47 ± 0.031	7.55 ± 0.006
Salinity (psu)	30.7	30.8	30.8	30.7	30.5	30.6	29.5	29.5	29.5	29.5	29.5	29.5
A_T_ (µmol kg^−1^)	2216	2208 ± 8.46	2218 ± 0.30	2215 ± 2.33	2195 ± 10.30	2186 ± 16.57	2126 ± 2.29	2153 ± 1.61	2120 ± 7.00	2155 ± 30.90	2166 ± 3.15	2167 ± 4.99
pCO_2_ (µatm)	926.28	902 ± 7.64	785 ± 15.76	1587 ± 221.71	1727 ± 24.79	1442 ± 11.08	726 ± 2.21	791 ± 64.24	583 ± 19.93	1732 ± 61.12	1689 ± 3.16	1414 ± 18.83
HCO_3_^−^ (µmol/kg)	1967.72	1955 ± 7.64	1934 ± 4.17	2011 ± 78.76	2049 ± 7.29	2018 ± 15.50	1849 ± 2.61	1888 ± 18.09	1795 ± 2.87	2017 ± 31.77	2024 ± 8.39	1998 ± 2.41
CO_3_^2−^ (µmol/kg)	102.45	104.17 ± 0.40	117.13 ± 1.84	63.01 ± 3.89	59.82 ± 1.28	69.42 ± 0.53	113.47 ± 0.02	108.86 ± 6.75	133.24 ± 4.12	56.63 ± 0.21	58.50 ± 2.13	68.08 ± 1.09
CO_2_ (µmol/kg))	26.92	26.14 ± 0.10	22.76 ± 0.45	45.99 ± 6.42	50.04 ± 0.71	41.78 ± 0.32	21.16 ± 0.06	23.06 ± 1.87	16.99 ± 0.58	50.45 ± 1.78	49.20 ± 2.20	41.18 0.54
NO_3_ (µM)	3.01 ± 0.22	5.13 ± 0.16	3.03 ± 0.97	3.50 ± 2.66	4.48 ± 1.37	3.05 ± 0.33	13.60 ± 0.10	12.86 ± 0.04	13.20 ± 0.03	13.12 ± 0.01	13.18 ± 0.64	13.06 ±
Si (µM)	9.82 ± 0.30	8.44 ± 0.55	6.49 ± 4.16	9.13 ± 2.99	8.26 ± 0.42	4.76 ± 0.12	10.91 ± 0.12	12.71 ± 1.58	11.25 ± 0.36	11.08 ± 0.97	10.82 ± 0.49	11.98 ± 2.00
PO_4_^3−^ (µM)	0.50 ± 0.02	0.06 ± 0.06	0.25 ± 0.007	0.59 ± 0.18	0.58 ± 0.12	0.45 ± 0.02	1.12 ± 0.09	1.27 ± 0.01	1.12 ± 0.12	1.11 ± 0.02	1.09 ± 0.11	1.06 ± 0.05

The values represent average (n = 2–3) ± SD.

Photosynthetic rates of juvenile *Macrocystis* sporophytes ranged from 4.55 to 19.73 µmol O_2_ L^−1^ g^−1^ h^−1^ but were unaffected by temperature, pCO_2_/pH, or location (Supplementary Table S3; Fig. 1). No difference in photosynthetic responses were observed across experimental trials (Table 1). The photosynthetic parameters estimated from the rapid light curves, including electron transport efficiency (α), maximum electron transport rates (ETR_max_) and saturating light (E_k_) were unaffected by temperature, location or pCO_2_/pH treatments (Table 1, Supplementary Tables S1 and S3). However, *Fv/Fm*, which is an established method to quantify temperature stress in macroalgae, was negatively affected by warmer temperature (*p* = 0.031) with the lowest values observed at 20 °C compared to those at 16 °C (Tukey test, *p* < 0.011). Moreover, differences in *Fv/Fm* and ETR_max_ among experimental trials were detected (Table 1, Supplementary Table S1).

**Figure 2:**
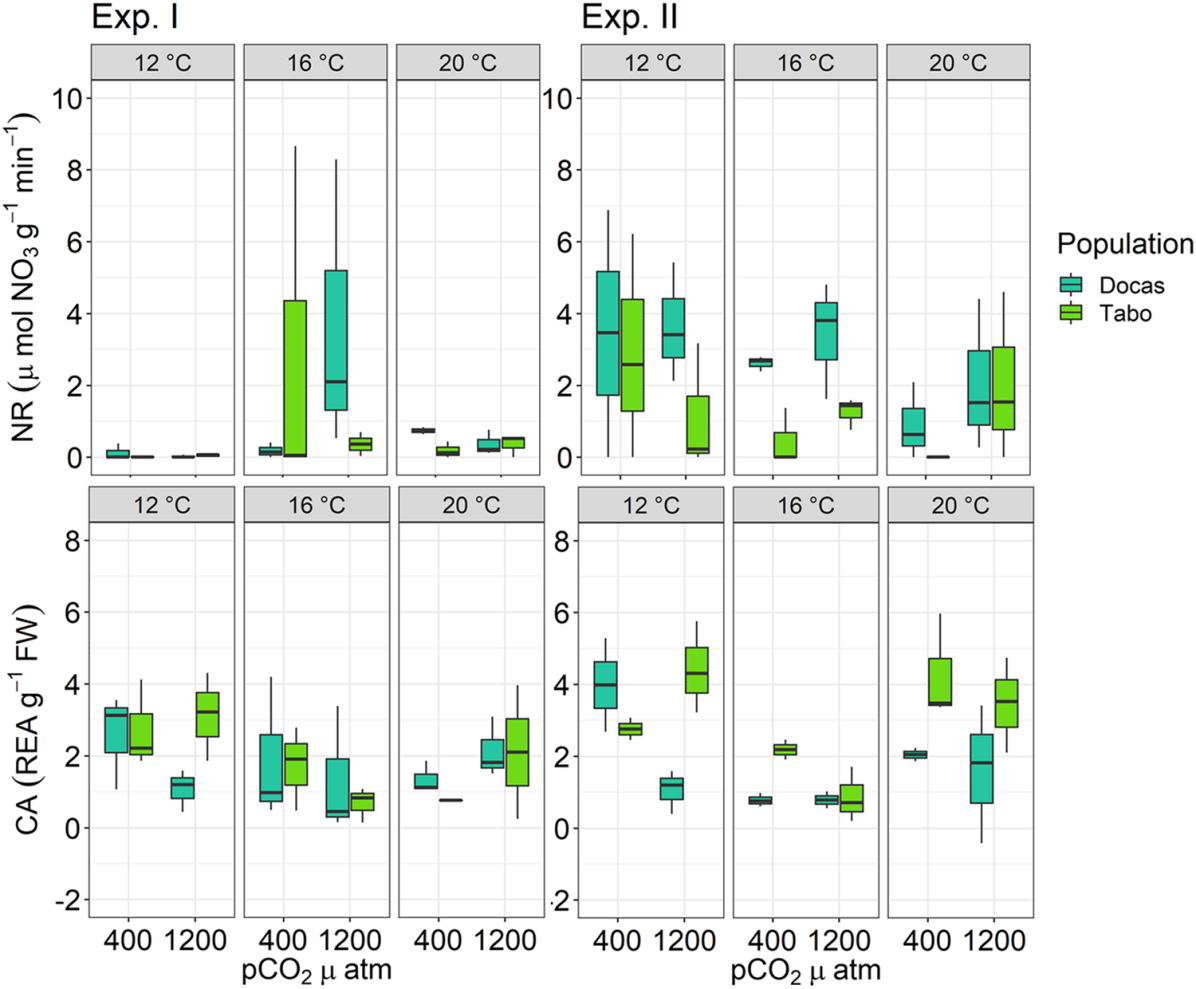
Nitrate reductase (NR) and Carbonic anhydrase (CA) activities of juvenile *Macrocystis* sporophytes from Las Docas and El Tabo incubated for three weeks (Experimental trial I and II) under three temperature (12, 16 and 20 °C) and two pCO_2_ treatments (~ 400 and 1200 µatm). Box-plots showing median size (line within box), 25th and 75th percentiles (ends of box), and minimum and maximum distribution of the data (whisker).

Nitrate reductase (NR) activity ranged from 0 to 8.28 nmol NO_3_^¬^ h^−1^ g^−1^ FW and varied among locations (*p* = 0.01, strongly upwelled site > weakly upwelled site, Fig. 2). While temperature, pCO_2_/pH or interactions had no effect on NR activity (Supplementary Table S3), NR activities differed across experimental trials (*p* = 0.0001, Table 1). Specifically, NR activities in Trial II individuals were higher than those in Trial I individuals but did not affect NR responses to temperature or pCO_2_ concentrations.

Carbonic anhydrase (CA) activity ranged from 0 to 5.71 REA g^−1^ FW and varied among temperature treatments (*p* = 0.001; Fig. 2) and locations (*p* = 0.008; weakly upwelling site > strongly upwelled site) (Table 1, Supplementary Table S3). CA activity was higher at 12 °C than at 16 °C (Tukey test, p = 0.036) but similar to CA activity at 20 °C (Tukey test, *p* = 0.1250). No differences in CA activities were observed across experimental trials (Table 1, Supplementary Table S3).

Photosynthetic pigments (Chl *a*, Chl *c*, fucoxanthin) all varied with all factors through several interactions (Table 1, Supplementary Table S3). The content of Chl *a* in juvenile *Macrocystis* sporophytes was affected by temperature, location and trial through two-way interactions (Table 1), but unaffected by pCO_2_/pH. Temperature affects appeared pre-eminent (through strong main effects [*p* = 6.352e^−07^; 12 °C > 16 °C < 20 °C] and interactions with location and trial (Table 1, Supplementary Tables S1 and S3). These interactions were mostly driven by a slightly higher Chl *a* content in individuals from the weakly upwelled site incubated at 20 °C, at either 400 or 1200 µatm, in Trial II compared to those from Trial I. The content of Chl *c* in juvenile *Macrocystis* sporophytes varied among locations (p = 0.002; weakly upwelled site > strongly upwelled site; Table 1) and experimental trials, and via a three-way interaction, but it was unaffected by pCO_2_/pH and temperature (Supplementary Tables S1 and S3). The Temperature × Location × Trial effect was mostly driven by a slightly higher Chl *c* content in individuals from the weakly upwelled site incubated at 20 °C at 400 µatm in Trial II compared to those from Trial I (Supplementary Table S1). Finally, the content of fucoxanthin was affected by temperature, location and trial through two-way interactions (Table 1), but unaffected by pCO_2_/pH (Table 1, Supplementary Table S3). The Temperature × Location × Trial effect was mostly driven by a slightly higher fucoxanthin content in individuals from the weakly upwelled site incubated at 12 °C under either 400 or 1200 µatm in Trial II compared to those from Trial I (Supplementary Table S1).

**Figure 3:**
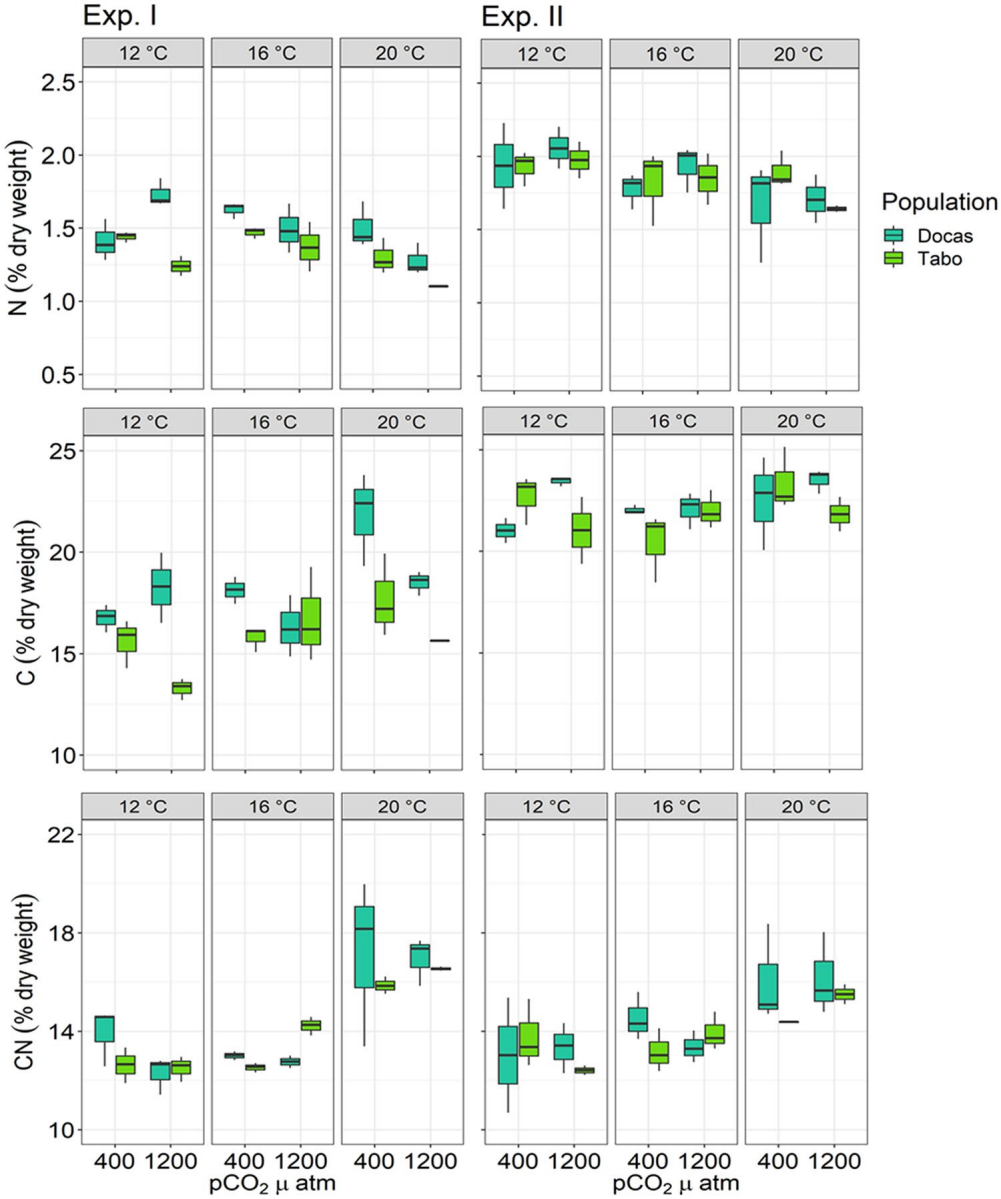
Total nitrogen and carbon content, and C/N ratios of juvenile *Macrocystis* sporophytes from Las Docas and El Tabo incubated for three weeks (Experimental trial I and II) under three temperature (12, 16 and 20 °C) and two pCO_2_ treatments (~ 400 and 1200 µatm). Box-plots showing median size (line within box), 25th and 75th percentiles (ends of box), and minimum and maximum distribution of the data (whisker).

The total internal N content in juvenile *Macrocystis* sporophytes ranged from 1.01 to 2.22% dry weight. N content varied among treatments (Table 1), with notable main effects of temperature (*p* = 9.710e^−05^; 12 °C = 16 °C > 20 °C) and location (*p* = 0.01728; strongly upwelled site > weakly upwelled site), but was unaffected by pCO_2_/pH (Supplementary Table S3; Fig. 3). These effects were mostly driven by higher N content in all individuals in Trial II compared to those from Trial I (*p* < 0.001) (Supplementary Table S3; Fig. 3). Similarly, total C content ranged from 12.70 to 25.13% dry weight, and varied among temperature treatments and locations (Table 1), but was unaffected by pCO_2_/pH (Supplementary Table S3; Fig. 3). Again, notable main effects were temperature treatments (*p* = 0.0008; 12 °C = 16 °C < 20 °C) and locations (*p* = 0.0001; strongly upwelled site > weakly upwelled site, Fig. 3). Total C content varied across experimental trials, with higher total C content in individuals from Trial II than those from Trial I (*p* =  < 0.0001) (Supplementary Table S3). The pCO_2_ × Temperature × Trial effect was driven by individuals from the weakly upwelled site incubated at 12 °C under 1200 µatm with lower total C content than those from both sites incubated at 20 °C under 400 µatm.

C/N ratio was strongly affected by temperature (*p* = 6.176e^−13^) but not by pCO_2_/pH, location nor interactions (Table 1, Supplementary Table S3; Fig. 3). The C/N ratio was higher (> 15) in individuals incubated at 20 °C compared to those incubated either at 12 °C (Tukey test, p = 0.008) or 16 °C (Tukey test, p = 0.04). No differences in C/N responses were observed across experimental trials (Table 1).

### Gene expression

**Figure 4:**
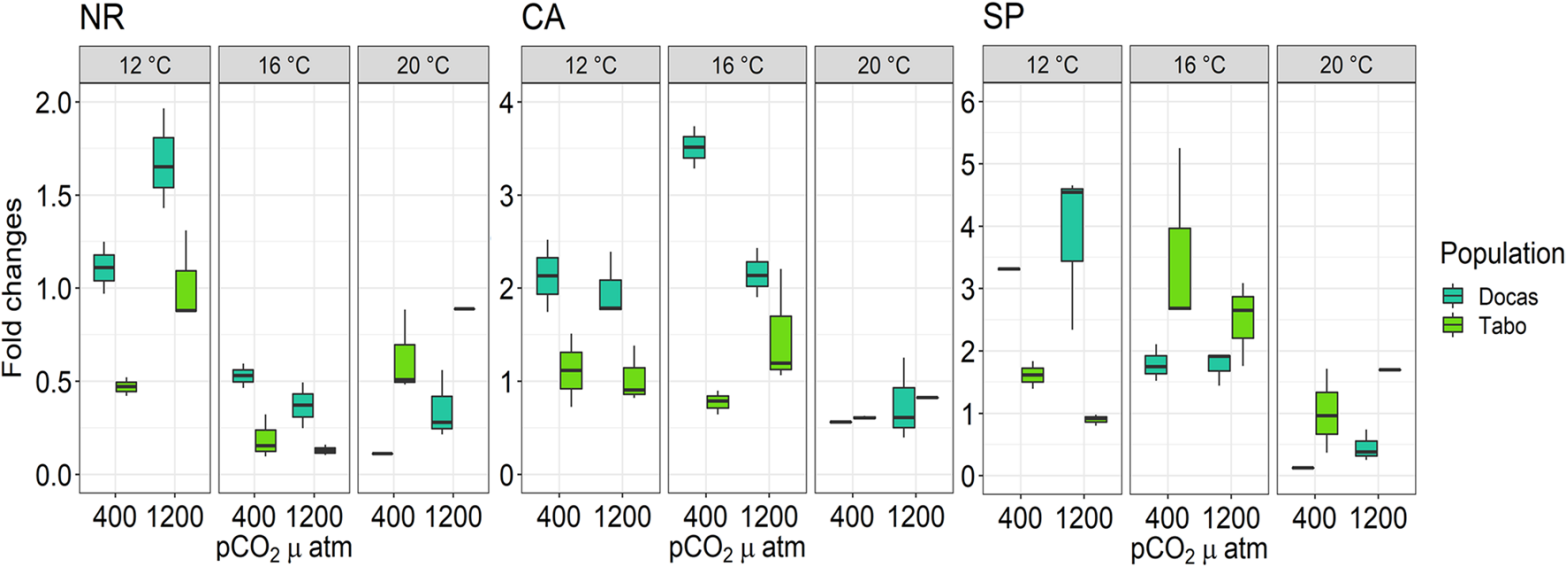
Relative expression levels (fold changes) of the nitrogen and carbon mechanisms related-genes, nitrate reductase (NR) and carbonic anhydrase (CA), and the stress related-gene, spermine (SP), from juvenile *Macrocystis* sporophytes (Las Docas and El Tabo) exposed to three temperature (12, 16 and 20 °C) and two pCO_2_ treatments (~ 400 and 1200 µatm) for three weeks. Box-plots showing median size (line within box), 25th and 75th percentiles (ends of box), and minimum and maximum distribution of the data (whisker).

Gene expression of nitrate reductase (NR) was increased by elevated pCO_2_/decreased pH (*p* = 0.008), decreased by increased temperature (*p* = 2.214e^−08^; 12 °C > 16 = 20 °C) and higher at the strongly upwelled site (location: *p* = 0.010; Table 1, Supplementary Table S3; Fig. 4). The pCO_2_ × Temperature effect on NR expression was driven by higher NR expression in individuals incubated at 12 °C under 1200 µatm compared to all other pCO_2_ × Temperature treatment combinations. The Temperature × Location effect was driven by higher NR expression at 12 °C in individuals from the strongly upwelled site than those from the weakly upwelled site.

Gene expression of carbonic anhydrase (CA) was decreased by increasing temperature and was higher at the strongly upwelled site (temperature: *p* = 1.918e^−05^; location: *p* = 3.747e^−06^; Fig. 4). The effect of pCO_2_/pH was expressed through two-way and a three-way interaction (Table 1, Supplementary Table S3). CA expression was greater in individuals grown at 16 °C than in individuals grown at 12 °C and 20 °C (Tukey test, *p* < 0.005; 12 °C < 16 °C > 20 °C). Moreover, individuals from the strongly upwelled site had higher CA expression than those from the weakly upwelled site (Tukey test, *p* = 0.008). The interaction of the main effects on CA expression (Table 1) was driven by higher CA expression in individuals from the strongly upwelled site incubated at 16 °C under 400 µatm compared to all other treatment combinations.

Gene expression of spermine/spermine synthase (SP) was only affected by temperature and the interaction between Temperature × Location (Temperature: *p* = 0.0002; temperature × location: *p* = 8.371e^−05^; Table 1, Supplementary Table S3; Fig. 4). SP expression was greater in individuals grown at 12 °C than that in individuals incubated either at 16 °C or 20 °C (Tukey test, *p* =  < 0.005). The interaction between temperature and location was driven by higher SP expression in juvenile sporophytes from the strongly upwelled site incubated at 12 °C compared to those from the weakly upwelled site (Tukey test, *p* = 0.008).

## Discussion

Our results show that *Macrocystis* physiological responses to OA and OW did not differ among distinct populations. Both populations were similarly (negatively) affected by elevated temperature (reduced growth) and mostly unaffected by OA. These results do not support our hypothesis that individuals from more fluctuating environments (strong upwelling) will be more tolerant to OA and high temperature. These results are opposed to previous studies on marine organisms (i.e., copepods, mussels, water fleas and calcifying algae), in which individuals from populations exposed to more fluctuating environments, i.e., temperature or CO_2_, showed greater tolerance to elevated temperature^72–74^ and OA, respectively^28,75,76^. Despite the lack of distinctly different responses to OA and OW, *Macrocystis* did show differences in molecular responses and physiological traits among populations. For example, growth and NO_3_^−^ assimilation (NR activity) as well as NR and CA gene expression were higher in individuals from the strongly upwelled site than those from the weakly upwelled site. Moreover, differences in gene expression patterns were also observed, supporting our second hypothesis. This might be driven by natural variability in nitrogen (i.e., NO_3_^−^), CO_2_ concentrations and SSTs between sites, suggesting that individuals from the strongly upwelled site might have greater C and N assimilation capacities than those from the weakly upwelled site, which might be attributable to phenotypic plasticity or local adaptation. However, further work including additional populations and fluctuating experimental regimes (rather than static conditions) are needed to precisely determine how natural variability^77^ might affect *Macrocystis* resilience to future environmental changes.

Marine organisms that inhabit the coast of Central and Northern Chile are naturally exposed to fluctuations in nutrients, SSTs and CO_2_/pH levels due to natural events such as upwelling^22,36,40,78^ and interannual variability associated with El Niño-La Niña cycles^79^. Previous studies have shown that this local exposure to supersaturated CO_2_ waters with reduced pH can ameliorate the negative effects of elevated CO_2_ concentrations on temperate calcifying invertebrates (mussels and sea urchins), showing greater tolerance to OA than individuals from less fluctuating environments^29,80^. One explanation for this is that these organisms may have developed physiological mechanisms to inhabit in these pH fluctuating areas, favouring its adaptation capacities to OA^81,82^. However, in calcifying algae, the role of natural variability on their physiological responses to OA have been more diverse and complex, mostly depending on their calcification mechanisms and internal chemistry regulation^75,83,84^. For example, a recent study on coralline alga has shown that exposure to greater variability in pH does not seems to play a key role in fostering its capacity to acclimate/adapt to OA^85^. Although few studies have evaluated how natural variability in CO_2_ might influence non-calcifying macroalgae responses to OA^63^, we found that elevated CO_2_ and reduced pH did not affect the physiological performance (growth and photosynthesis) of juvenile *Macrocystis* sporophytes from distinct populations. This may be attributed to photosynthetic mechanisms developed by the species. *Macrocystis* possess effective carbon concentrating mechanisms (CCMs), depending mainly on HCO_3_^−^ as the main inorganic carbon (Ci) source to support their photosynthesis that might be saturated at the current ambient Ci conditions^86^. Therefore, increased CO_2_ concentration does not greatly affect *Macrocystis*’ physiological performance, which corroborates previous studies in this species^86,87^ and other kelps (i.e. *Saccharina latissima* and *Laminaria solidungula*)^88^. However, we do not discard the possibility that this effect might differ across different kelp life stages depending on their physiological requirements and inorganic carbon physiology^63,89,90^.

Ocean acidification can also have direct effects on other physiological processes such as nitrogen uptake and assimilation, usually attributed to supporting greater metabolic rates (e.g., growth) under elevated CO_2_ concentrations^91,92^. Previous studies have shown that the physiological activity of NR can be up-regulated under OA conditions in non-calcifying (i.e. *Ulva sp*., *Macrocystis pyrifera*)^92,93^ and calcifying algae (i.e. *Corallina officinalis*)^91^. However, to our knowledge, this is the first study assessing the effects of OA on the expression of NR gene in *Macrocystis*, and possibly in kelps. Although we did not observe any change in the physiological NR activity across experimental treatments [only among populations (ω^2^ = 6.21%) and trials (ω^2^ = 16.49%), which might be explained by the small differences in SW nitrate concentrations], NR gene expression was higher under OA conditions, and differed markedly among populations (ω^2^ = 3.7%) and temperature (ω^2^ = 53.7%). Similarly, other genes of the N assimilation pathway (i.e., Nitrite reductase, NiR) has been upregulated in marine diatoms (i.e., *Phaeodactylum tricornutum*) under OA and attributed to maintaining internal pH homeostasis under acidified conditions^94^. As we did not observe any changes in photosynthesis or growth under OA, enhanced NR expression might be associated with the effects of elevated CO_2_ on NR novo synthesis rather than on C assimilation^92,93^. This suggests that the expression of the constitutive NR might be a genetically regulated process and be part of the adaptation to fluctuating environments in NO_3_^−^ (strongly upwelled site > weakly upwelled site). Therefore, the differences observed in NR expression among populations, especially at 12 °C (T × P: ω^2^ = 17.8%), can represent different adaptive capacities, indicating that the exposure to cold NO_3_^−^-enriched SW driven by upwelling might have influenced the nitrogen assimilatory process in individuals from the strong upwelling site.

The expression patterns of metabolic-related genes in individuals of *Macrocystis* have been previously described along its depth distribution (0–18 m), showing that metabolic genes can be up or down-regulated depending on environmental conditions (e.g., nitrate, light and temperature)^95^. Similar to our results, higher expression of NR has been associated with higher NO_3_^−^ concentrations, but also with previous nitrate exposure, which can support our results. However, the expression of SP, which has been mostly studied in plants and associated with temperature stress responses^96^, showed differential responses among distinct populations. We found higher expression of SP at 12 °C in individuals from the strongly upwelled site but at 16 °C in individuals from weakly upwelled site, with a notable interactive effect between temperature and population (ω^2^ = 37.8%). Konotchick et al. (2013) showed that the expression of SP in individuals of *Macrocystis* was higher in winter than in summer, suggesting that the expression of SP genes might be associated with metabolic adjustments to low temperatures rather than to high temperatures. This might explain, at least in part, the differences observed among populations in our study, where individuals from the strongly upwelled site are usually exposed to colder waters driven by upwelling events than those from the weakly upwelled site. Thus, individuals from the strongly upwelled site might have greater adaptive capacities to compensate for low temperatures than those from the weakly upwelled site. However, it is difficult to determine if other environmental factors are also regulating the expression of this metabolic gene as it has been poorly studied in juveniles. Therefore, further work coupling physiological studies and molecular responses are needed to clarify its metabolic role in temperature stress responses.

Temperature and inorganic nitrogen play critical roles in macroalgae physiology and ecology, controlling key physiological processes such as photosynthesis and growth^97–99^. Therefore, it is not surprising that *Macrocystis’* physiological and molecular responses were more strongly influenced by temperature rather than OA, at least over short-term exposure and under the OA scenario projected by 2100. However, long-term exposure to OA conditions may exacerbate the negative impact of elevated temperature on other life stages (microscopic)^100^. Growth, the maximum quantum yield of PSII (*Fv/Fm*), tissue N content, C/N ratio, and NR and CA gene expressions of juvenile *Macrocystis* sporophytes were negatively affected by OW (20 °C treatment). Our results shown that the main effect of temperature can explain more than 30% of variance of growth, C/N ratio, NR and CA gene expression, while OA explain less than 1%. Previous studies have shown the negative impact of elevated temperatures on growth rates of *Macrocystis*^101^ and other kelp species (i.e., *Laminaria digitata* and *Laminaria ochroleuca*); negative effects on *Fv/Fm* have also been observed^102,103^. The negative impact of elevated temperature on some species can be closely related to the thermal optimum for growth and other temperature-dependent physiological traits. It is generally thought that *Macrocystis* tends to be a more cold-adapted species, and cannot survive at temperatures above 20 °C^53,63^. A recent study has shown that the optimum temperature (T_opt_) for growth in adult’s individuals of *Macrocystis* is close to 16 °C, and temperature above T_opt_ (i.e., at 24 °C) can negatively affect its physiological performance^104^. However, NO_3_^−^ enrichment can modulate these responses, enhancing for example, their physiological thermal tolerance, ameliorating the negative impacts of sub-optimal temperatures^104^. Although we found that both populations were equally negatively affected by elevated temperatures, relative growth rates remained above 9% day^−1^, which might be attributed to the non-limiting level of NO_3_^−^ supplied during the experiments (> 5 µM). Contrary to the growth rates, photosynthetic rates were unaffected by elevated temperatures, which might be explained by its higher capacity to acclimate to increases in temperature^99,105^. Moreover, higher C/N ratios (> 15) in individuals grown at 20 °C from both populations suggest that internal nitrogen reserves were likely utilized to increase photosynthetic rates at high temperatures^106^. Similar to our results, Sanchez-Barredo et al.^103^ have shown that juvenile *Macrocystis* photosynthetic performance is almost unaffected by elevated temperature, indicating that other environmental changes (e.g., reduced light) can be more detrimental for juveniles *Macrocystis* sporophytes than thermal stress.

In conclusion, our results showed that temperature, rather than OA, is a much stronger driver controlling juveniles *Macrocystis* performance, at least over short-term exposure, and that intrinsic differences among populations influence their responses to low and optimum temperatures and to elevated CO_2_ (gene expression). Local exposure to cold NO_3_^−^-enriched SW (coastal upwelling) can enhance *Macrocystis* tolerance to fluctuation in temperatures, but mostly to lower temperatures (12 °C) rather than elevated temperatures (at least under static experimental conditions). It is also important to note that the molecular responses observed in the metabolic-related genes indicate clear differences in the adaptive capacities among populations that can be of great importance to determine the species responses to climate change (e.g., most effects were explained by temperature). However, further work is needed to clarify whether these differences are driven by the natural variability in NO_3_^−^, temperature and CO_2_ variability driven by coastal upwelling. Currently, little is known about how these adaptive differences can modulate kelp’s responses to warming and other local changes such as eutrophication, as most recent studies have focused on determining the effects of local adaptation to pH/CO_2_ regimes in calcifying organism’s responses to OA^107^. Moreover, further studies including early life stages are urgently needed as some of life stages can be more vulnerable to environmental changes driven by global change, and therefore, negatively impact kelp forest recruitment and population persistence.

## Material and methods

### Study sites

In February 2018, fertile sporophylls of *Macrocystis* were collected from sites naturally exposed to different regimes of upwelling (strong or weak) from central Chile (~ 33°S). This region is dominated by seasonal upwelling, and spatial variation in SSTs alongshore, during strong southerly winds and active upwelling, is easily observed in thermal imagery (see e.g., Broitman et al.^43^; Wieters et al.^32^; Navarrete et al.^31^). Within the region, two locations were selected: Las Docas (33°08´S, 71°42´W), which is closely located to the major upwelling center: Punta Curaumilla, and El Tabo (33°27´S, 71°66´W), which is weakly or less influenced by upwelling^31,32,36,38,108^ (Supplementary Figure S1). Variability in the intensity (strong or weak) of upwelling occurring in our study area has been previously described from thermal variations, using time series of in situ SSTs in shallow near shore waters (see Fig. 1 in Navarrete et al.^31^). Moreover, onshore nutrient concentrations are tightly correlated with temperature in the study area^32,34,42^. While the upwelling area is characterized by cold (< 14 °C), low dissolved oxygen, high salinity (> 34 psu) and nutrient-rich conditions (phosphate > 2.0 µM; nitrate > 15 µM; silicate > 10 µM) during an upwelling event^33^, less influenced upwelling sites (e.g., El Tabo) exhbited higher temperaures and nutrient-poor waters during spring–summer upwelling season^32,34^.

### Sporophyll collection and meiospore release

At each of the two locations, five to seven mature sporophylls were collected from each of twelve adult sporophytes of *Macrocystis*. In the laboratory, collected sporophylls were gently brushed and cleaned of visible epibiont using filtered seawater (0.2 µm), blotted dry, wrapped in tissue paper and stored overnight at 4°C^109^. To induce meiospore release, sporophylls from each population were pooled and immersed in 0.2 µm-filtered seawater (SW) at room temperature (~ 16 °C) for 15–30 minutes^110^. The obtained meiospore suspensions were used to start the experimental cultures. Initial meiospore density was determined using a 0.1 mm depth Neubauer cell-counting chamber. Cultures were initiated by inoculating 1.5–2.0 mL of meiospore suspension (25,000 spores cell ml^−1^) into sterile plastic bags containing 300 mL of filtered SW enriched with Provasoli medium (n = 10, for each population) in order to obtain a substantial number of juveniles in a short time^111,112^. Simultaneously, small Petri dishes (Ø = 4 cm), containing 25,000 meiospore cell mL^−1^, were also inoculated to monitor the early life stages development (e.g., Leal et al.^90^).

After eight days of culture, 100 µL of GeO_2_ (0.04 g l^−1^) was applied to avoid overgrowing of diatoms^113^. Culture medium was exchanged once per week and the early development of gametophytes was followed by taking photographs from the small Petri dishes under an inverted microscope (Olympus CKX41).

Meiospores were cultured for two months in a temperature-controlled room at 12 °C, with a photoperiod of 16:8 L:D and 45 ± 5 µmol photons m^−2^ s^−1^ which is within the range of saturating light intensities (40–70 µmol photons m^−2^ s^−1^) described for kelp gametophytes and embryonic sporophytes^114^. Light intensity was provided overhead by white LED tubes (T8, 165–265 V 50/60 Hz) and measured using a Li-COR 250 light meter. When juvenile sporophytes became visible to unaided eyes, they were easily detached from the plastic bags and transferred to 1 L Erlenmeyer flasks with further expansions to 2, 5 and 20 L in a free floating system^111^. Culture medium was exchanged once a week, and flasks were vigorously aerated and incubated under the same controlled experimental conditions described above. After reaching a length of 1–2 cm, juvenile sporophytes were transferred to a CO_2_ mesocosm and used for further experiments.

### Experimental design

Juvenile sporophytes from the two populations were exposed for 21 days to a combination of two pCO_2_ concentrations (ambient SW with 400 µatm and pH 7.9 and a OA scenario with 1200 µatm and pH 7.5) and three temperature treatments (12 °C, 16 °C and 20 °C that simulated winter, summer and OW SSTs, respectively). Each experimental treatment contained three independent replicates of 5-L acrylic tanks (total n = 36). Five juvenile sporophytes of each population were placed into a randomly selected 5-L acrylic tank containing either ambient or CO_2_-modified SW, with three replicates for each population. We replicated the experiment through time from June to July 2018 with a total of two trials: experiment I and experiment II, respectively. While SW nutrient concentrations might have played a role in the organism’s responses (e.g., physiological stress associated with nitrogen limitation), this was likely minimal since in both experiments inorganic nitrogen concentrations (i.e., NO_3_^−^) remained above the growth limiting concentrations described for *Macrocystis* (1–2 µM)^115^ (Table 2). Despite the fact that experimental trials were properly random effects (out of our scope of inference), we treated them as fixed factors to avoid numerical problems as suggested by Hodges (2016)^116^ (*see Statistical analyses*).

Three thermo-regulated baths were prepared at each experimental temperature (12, 16 and 20 °C) containing a total of twelve 5 L-acrylic culture tanks (n = 6 for each CO_2_ treatment). Juvenile sporophytes were gradually acclimated from the pre-experimental temperature (12 °C) to the experimental temperatures (16 °C and 20 °C), using a ramping approach with changes of 2 °C day^−1^ to avoid physiological stress. Light intensity was provided over each thermo-regulated bath by LED tubes (T8 165–265 V 50/60 Hz, JIE) providing a light intensity of 45 ± 5 µmol photons m^−2^ s^−1^ set to a 16:8 L:D photoperiod regime. Temperature and light conditions were monitored continuously using HOBO pendant temperature/light data loggers (HOBO, 64K-UA-002-64, MA, USA). The CO_2_/pH level of the OA treatment was achieved using a CO_2_ mesocosm described in detail in Navarro et al.^117^ and Torres et al.^118^. In brief, for the ambient treatment (400 µatm), pure atmospheric air was bubbled into three header tanks containing ambient filtered SW (1000 L) maintained at each experimental temperature. For the OA treatment dry air was blended with pure CO_2_ to the target CO_2_ concentration (1200 µatm) using mass flow controllers (MFCs, Aalborg Instruments & Controls, Inc.) for air and CO_2_. Ambient or modified-CO_2_ SW flowed into each of the twelve 5 L-acrylic culture tanks near the top of the tank and exited the tank via an outflow pipe that was located close to the bottom of the tank (water flow ~ 0.3 L min^−1^). Also, each culture tank was aerated with the air or CO_2_/air mix using sterile serological pipettes (25 mL) to ensure continuous mixing and turbulence to maintain the sporophytes in permanent flotation. Water samples were randomly taken from header and cultures tanks for monitoring pH, alkalinity and seawater carbonate parameters, nutrient concentrations and salinity were measured at the start of the experiment (Table 2) and pH was monitored throughout the experiment by randomly taking SW samples from header and culture tanks (Supplementary Table S2).

### Physiological traits and biochemical composition

Photosynthetic rates expressed as oxygen evolution were measured on the last day of the temperature/CO_2_ experiments, for each of the 36 experimental units (n = one juvenile per experimental unit). To do this, a single juvenile sporophyte of approximately 0.3–0.5 g fresh weight (FW) was incubated separately in a glass vial containing either 20 ml of ambient or CO_2_-modified SW. Thermo-regulated baths were prepared for each experimental temperature (12, 16 and 20 °C), and placed on the top of an orbital shaker table (Labnet, International, Inc., Woodbridge, USA) set at 100 rpm to provide water movement and minimize concentration boundary layer effects. Oxygen evolution was measured at the start of the incubation period and after 1 h, using a Presens 50 µm oxygen microoptode (Microx 4, PreSens, Germany). Photosynthetic rates were measured under a saturating light intensity of 45 ± 3 µmol photons m^−2^ s^−1^ that was provided overhead by LED tubes. Photosynthetic rates were determined from the initial and final oxygen concentrations (µmol L^−1^), and standardized to algal wet weight (g) and incubation volume (L).

After measuring photosynthetic rates, Chlorophyll *a* fluorescence of photosystem II was measured using a junior Pulse Amplitude Modulation fluorometer (PAM, Walz, Germany). Single juvenile sporophytes from each of the 36 experimental units (n = one juvenile per experimental unit) were dark-adapted for 20 min before exposure to the PAM’s photosynthetic active radiation (PAR, 0–422 μmol photons m^−2^ s^−1^). Rapid Light Curves (RLCs), relative Electron Transport Rate (rETR) versus irradiance, were conducted immediately following dark adaptation. Calculations of rETR of algal samples were estimated using the equation: $rETR=Y\left(II\right)\times \mathrm{EPAR }\times \mathrm{A }\times 0.5$, where Y(II) is the quantum yield of photochemical quenching, E the incident irradiance of PAR, A the average ratio of light absorbed by algal tissue (0.8 for kelps) and 0.5 is the factor assuming that half of the electrons required to assimilate on CO_2_ molecule are supplied by PSII^119^. The rETR-RLCs were fitted according to^120^ and saturating irradiance (E_k_), initial slope (α) and the maximum rETR were calculated from each RLC. The optimum quantum yield (*Fv/Fm*), which represents a good indicator of maximal algal photosynthetic efficiency^121^, was calculated right after dark adaptation.

Relative growth rates (RGRs, % day^−1^) were estimated after measuring photosynthetic rates and Chlorophyll *a* fluorescence by the difference in the initial and final algal FW (g) (n = 5 juveniles per experimental unit), after three weeks of incubation, using the formula:

$$RGR={\rm{ln}}\left(\frac{Wt}{W0}\right)\times t-1 \times 100$$

where W_0_ is the initial FW and W_t_ is the final FW after *t* days of incubation. The FW was estimated after blotting off surface water with tissue paper.

After measuring photosynthetic and growth rates, juvenile sporophytes (n = 5 per experimental unit) were immediately frozen in liquid N_2_ and stored at – 80 °C to assess nitrate reductase (NR) (0.20–0.30 g), carbonic anhydrase (CA) (0.10–0.15 g), pigment concentrations (0.15–0.20 g) (chlorophyll *a*, *c* and fucoxanthin), the expression of metabolic-related genes (NR, CA) and a metabolic stress response gene (Spermine, SP) (0.10–0.20 g). Tissue samples for total C and N content (0.008–0.010 g) determinations were oven dried for 48 h at 60 °C. Total internal C and N contents on a dry weight (DW) basis were determined using a CNHS-932 elemental analyser.

Nitrate reductase activity was measured by nitrite production in an in vitro assay^122^. NR extraction methodology is described in detail in Fernandez et al.^93^. Briefly, frozen samples were ground to a fine powder using liquid N_2_, and NR was extracted in a 200 mM Na-Phosphate buffer (pH 7.9), containing 3% w/v BSA, 0.3% w/w polyvinylpyrrolidone (PVP), 2 mM Na-EDTA and 1% w/v Triton X-100 (all Sigma, St Louis, MO, USA). The enzymatic reaction was carried out in a 200 mM Na-Phosphate buffer (pH 7.9), 0.2 mM NADH, 220 µL of homogenized extract, and 100 mM KNO_3_^–^ added to start the reaction at 12 °C. The reaction was stopped after 20 min by adding 1 M zinc acetate and the concentration of NO_2_ formed was measured spectrophotometrically as described by Strickland and Parsons^123^. The NR was expressed as µmol NO_3_ g^−1^ FW min^−1^.

Carbonic anhydrase activity was measured using the method described in detail in Fernandez et al.^124^. Briefly, frozen samples were ground to a fine powder using liquid N_2_, and CA was extracted in a 50 mM Tris buffer (pH 8.5), containing 2 mM DTT, 0.3% PVP, 5 mM Na-EDTA and 15 mM ascorbic acid. CA activity was measured potentiometrically at 0–2 °C by measuring the time for a linear drop in pH of 0.4 units from 8.3 to 7.9. pH and temperature were simultaneously measured using a ROOS electrode (Orion8107BNUMD, CA, USA) coupled to Orion 3-Starts Plus pH Benchtop meter (Orion, Thermo Scientific, CA, USA). The relative CA activity was determined using the formula:

$$REA=\frac{Tb}{Ts} -1$$

where Tb and Ts represent the times in seconds required to drop by 0.4 pH units in the uncatalyzed reaction (Tb, buffer without algae) and in the enzyme-catalyzed reaction (Ts), respectively. REA was standardized to the sample’s FW (REA g^−1^ FW).

The content of photosynthetic pigments (chlorophyll *a*, *c* and fucoxanthin) was analysed using the methods described in Seely et al.^125^ and Wheeler et al.^126^. Briefly, a known amount of frozen biomass of ~ 0.2–0.4 g FW of each individual was placed in test tubes. First 4 ml of dimethyl-sulfoxide (DMSO) was added and left to extract for 10 min. The DMSO extract was poured off and absorption was measured with an infinite 200 PRO microplate reader (Nanoquant, Tecan Group Ltd, Männedorf, Switzerland) at wavelengths of 665, 631, 582, and 480 nm. Immediately after, 6 ml of 90% acetone (v/v) was added to the tissue and left to extract for 30 min. During this time, the test tubes were occasionally agitated. After 30 min, the 90% acetone extract was poured off and absorption of the extract was measured at wavelengths 664, 631, 581 and 470 nm. Visual observation showed no remaining pigments in the seaweed tissue. Concentration of pigments was calculated using the equations given by Seely et al.^125^.

### Seawater analyses

Nutrient concentrations: nitrate, nitrite, silicic acid and phosphorus were analysed following the methods described in Strickland and Parsons^123^. Total pH (pH_T_) was measured in a closed 25 ml cell, thermostatically controlled at 25 °C, using a Metrohm 713 pH meter calibrated with Tris buffer (pH = 8.089) at 25.0 °C. Total alkalinity (A_T_) was determined by potentiometric titration in an open cell, according to Haraldsson et al.^127^. The pH_T_, A_T_ and salinity were used to calculate the rest of the seawater carbonate chemistry parameters, using CO2SYS software^128^ for experiments I and II (Table 2).

### RNA extraction and qPCR

To evaluate molecular responses of *Macrocystis* to the combined effect of temperature and CO_2_/pH, gene expressions of metabolic related genes were analyzed using juvenile sporophytes collected at the end of Experiment I. The oligonucleotides carbon-related gene, carbonic anhydrase (CA: F-ccagcggtgtacatgagaga and R-gccttttccacgctgactac), nitrogen-related gene, nitrate reductase (NR: F-ccggagaaggtcgatgct and R-cttatccctggggtcgatct) and stress-related gene, spermine/spermine synthase (SP:F-aatgcctatggcttcacctg and R-ggtaggaaggcacggttgta) were analysed (Primers created by^95^).

Juvenile sporophytes (*c.* 100–200 mg) were ground to a fine powder under liquid N_2_, using a pestle and mortar. All glassware and consumables were treated with RNase Away reagent (Invitrogen, Thermo Fisher Scientific, MA, USA). Total RNA and DNA were extracted using Pure Link RNA Mini Kit (Ambion, Life Technologies, CA, USA), according to the manufacturer’s protocols. After extraction, a treatment with RQ1 RNase-free DNase (Promega, WI, USA) was performed to eliminate residual genomic DNA. Nucleic acid concentrations were measured in a Qubit 3.0 (Invitrogen, CA, USA) using Quant-iT RNA assay kit for RNA and Quant-iT dsDNA HS assay reagents (Invitrogen, Life Technologies, CA, USA), and RNA quality was checked on a 1.5% agarose gel stained with ethidium bromide. cDNA was synthesized using a Revert Aid RT kit (Thermo Fisher Scientific, MA, USA). For each gene, the qPCR reactions were performed in a StepOnePlus™ Mastercycler (Applied Biosystems, Life Technologies, CA, USA) with Maxima SYBER Green/ROX qPCR master mix (Thermo Fisher Scientific, MA, USA) following the manufacturer´s protocol. Briefly, samples were incubated 10 min at 95 °C, followed by 40 cycles of 15 s at 95 °C and 60 s at 60 °C. Each sample was technically triplicated. We quantified expression levels using qPCR via the 2^−∆∆CT^ according to Livak and Schmittgen^129^, using the 18S mat (protein required for 18S rRNA maturations) gene as housekeeper for normalization and individuals not treated from each population as controls.

### Statistical analysis

All statistical analyses were performed using the R software version 3.5.1^130^. Prior to analyses, normality and homogeneity of residuals were verified by visual inspection of Q-Q- plots and histograms, and validated by the Shapiro–Wilk´s and Levene’s test, respectively. Data were transformed either by log10 or Box-Cox transformations^131^ to satisfy assumptions for parametric tests (ANOVAs). For all response variables (e.g., photosynthesis, growth, enzyme activities), linear models (lm function of R) were fitted with temperature, pCO_2_, location and experimental trial as fixed effects. The best fit model (with or without interactions among effects) was selected based on the second-order Akaike’s Information Criterion (AICc)^132^ score (Akaike’s^133^ criterion corrected for small sample sizes). Significance of fixed factors and their interactions was determined using the *anova* function of R software. When differences were significant at the *p* < 0.05 level, a posteriori Tukey’s test was performed (*multcomp* function). For gene expression data, linear models were fitted to analyse the effects of temperature, pCO_2_ and population on CA, NR, and SP expression. The best fit model (with or without interactions among effects) was selected based on the smallest AICc score, and when differences were significant at the *p* < 0.05 level (*anova* function of R), a posteriori Tukey’s test was performed. For all response variables, the magnitude of effects (omega squared: ω^2^) (proportion of the variance explained) were calculated for all fixed effects and interactions according to Graham and Edwards^134^ using R package “sjstats”^135^ (See Supplementary Table S3).

## Supplementary Information

Supplementary Information.
