# Supplementary material for: Effect of environmental history on the habitat-forming kelp Macrocystis pyrifera responses to ocean acidification and warming: a physiological and molecular approach
Source: Sci Rep. 2021 Jan 28;11:2510. doi: 10.1038/s41598-021-82094-7 (PMC7843619; doi:10.1038/s41598-021-82094-7)
Supplement: Supplementary file 1 — Supplementary Information. [file 41598_2021_82094_MOESM1_ESM.pdf]

Supporting information

Effect of environmental history on the habitat-forming kelp *Macrocystis pyrifera* responses to ocean acidification and warming: a physiological and molceular approach

Pamela A. Fernández, Jorge M. Navarro, Carolina Camus, Rodrigo Torres, Alejandro H. Buschmann

This PDF file includes:

- Figure S1
- Table S1 to S3

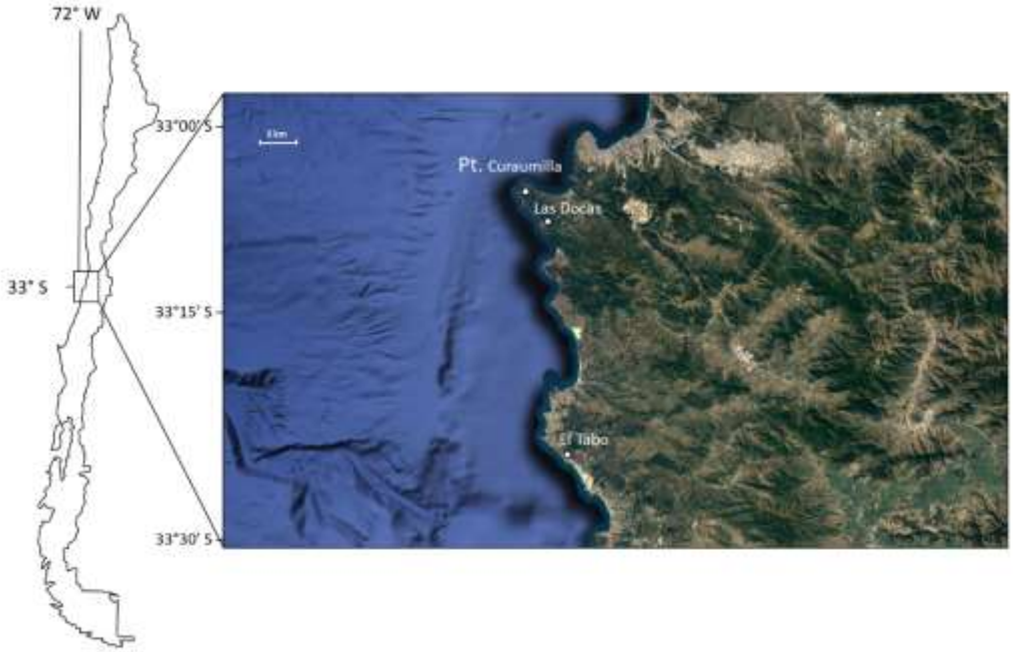

Supplementary Figure S1: Map of the coastline of Chile, showing the study area from central Chile (~33°S): Las Docas (33°08´S, 71°42´W), which is closely located to the major upwelling center: Punta Curaumilla, and El Tabo (33°27´S, 71°66´W). Image generated from the website map maker (URL: https://maps.co/gis/).

Supplementary Table 1: Chlorophyll *a* fluorescence parameters (α, rETRmax, Ek, Fv/Fm) and pigments content (Chl *a*, Chl *c*, Fx) in juvenile's sporophytes of *Macrocystis* collected at the end of each temperature/CO_2 experiments. The values represent average (n = 3) ± SD.

		Experimental trial I						***Experimental trial II***					
**Parameter**	**Location**	**Ambient pCO_2**			**OA scenario**			**Ambient pCO_2**			**OA scenario**		
		**12 °C**	**16 °C**	**20 °C**	**12 °C**	**16 °C**	**20 °C**	**12 °C**	**16 °C**	**20 °C**	**12 °C**	**16 °C**	**20 °C**
Alfa	Docas	0.40 ± 0.05	0.49 ± 0.04	0.34 ± 0.07	0.50 ± 0.06	0.44 ± 0.06	0.51 ± 0.12	0.43 ± 0.14	0.43 ± 0.12	0.34 ± 0.10	0.36 ± 0.11	0.486 ± 0.03	0.34 ± 0.03
	Tabo	0.38 ± 0.09	0.38 ± 0.03	0.37 ± 0.07	0.40 ± 0.06	0.42 ± 0.02	0.45 ± 0.06	0.43 ± 0.02	0.43 ± 0.04	0.45 ± 0.04	0.41 ± 0.14	0.32 ± 0.06	0.33 ± 0.10
rETRmax	Docas	132.8 ± 48.6	132.5 ± 11.1	141.2 ± 29.5	117.4 ± 27.6	182.7 ± 70.3	142.3 ± 55.5	123.3 ± 33.7	171.9 ± 63.2	139.7 ± 40.0	177.3 ± 63.4	148.9 ± 45.0	119.6 ± 29.2
	Tabo	209.56 ± 73.7	238.0 ± 27.17	127.8 ± 26.5	203.7 ± 23.7	179.6 ± 18.7	138.1 ± 84.8	152.9 ± 70.6	91.1 ± 34.5	126.2 ± 38.3	109.5 ± 20.4	184.3 ± 69.1	94.78 ± 56.7
Ek	Docas	347.61 ± 176.6	267.0 ± 14.4	423.2 ± 92.7	241.1 ± 78.9	406.6 ± 135.4	302.3 ± 179.0	308.1 ± 128.1	418.4 ± 152.3	410.2 ± 63.1	614.9 ± 483.1	305.7 ± 90.2	354.9 ± 118.9
	Tabo	543.2 ± 120.6	613.7 ± 29.87	360.7 ± 139.2	518.9 ± 132.2	417.9 ± 19.8	330.1 ± 253.	360.9 ± 177.2	208.3 ± 65.5	276.3 ± 70.0	286.2 ± 85.1	607.7 ± 337.7	270.7 ± 73.0
Fv/Fm	Docas	0.62 ± 0.03	0.64 ± 0.02	0.55 ± 0.05	0.63 ± 0.03	0.63 ± 0.02	0.62 ± 0.02	0.57 ± 0.09	0.61 ± 0.02	0.55 ± 0.10	0.55 ± 0.11	0.64 ± 0.01	0.57 ± 0.04

	Tabo	0.58 ± 0.07	0.60 ± 0.07	0.55 ± 0.03	0.59 ± 0.04	0.60 ± 0.009	0.60 ± 0.04	0.55 ± 0.02	0.58 ± 0.05	0.61 ± 0.01	0.54 ± 0.01	0.57 ± 0.05	0.54 ± 0.10
Chl a	Docas	0.202 ± 0.014	0.194 ± 0.05	0.228 ± 0.007	0.191 ± 0.017	0.241 ± 0.03	0.185 ± 0.100	0.174 ± 0.040	0.087 ± 0.04	0.252 ± 0.024	0.198 ± 0.030	0.146 ± 0.002	0.320 ± 0.09
	Tabo	0.196 ± 0.085	0.169 ± 0.03	0.207 ± 0.018	0.167 ± 0.023	0.182 ± 0.01	0.161 ± 0.001	0.302 ± 0.037	0.146 ± 0.03	0.361 ± 0.124	0.289 ± 0.026	0.141 ± 0.004	0.187 ± 0.05
Chl c	Docas	0.038 ± 0.004	0.035 ± 0.004	0.034 ± 0.005	0.035 ± 0.002	0.039 ± 0.003	0.031 ± 0.013	0.029 ± 0.005	0.034 ± 0.001	0.043 ± 0.010	0.036 ± 0.013	0.032 ± 0.007	0.061 ± 0.01
	Tabo	0.037 ± 0.017	0.032 ± 0.009	0.040 ± 0.006	0.030 ± 0.004	0.049 ± 0.005	0.033 ± 0.001	0.065 ± 0.003	0.042 ± 0.004	0.083 ± 0.035	0.087 ± 0.026	0.035 ± 0.007	0.040 ± 0.008
Fx	Docas	0.090 ± 0.005	0.089 ± 0.02	0.112 ± 0.006	0.089 ± 0.010	0.116 ± 0.02	0.084 ± 0.05	0.073 ± 0.019	0.074 ± 0.003	0.118 ± 0.015	0.086 ± 0.017	0.079 ± 0.011	0.151 ± 0.045
	Tabo	0.091 ± 0.037	0.080 ± 0.01	0.101 ± 0.008	0.079 ± 0.008	0.081 ± 0.01	0.104 ± 0.002	0.155 ± 0.017	0.101 ± 0.004	0.166 ± 0.049	0.136 ± 0.017	0.077 ± 0.003	0.086 ± 0.016

Supplementary Table 2: Seawater pH_T measurements throughout the temperature/CO_2 experimental trials (I-II). HT: Header tank.

Date	ID Culture tank (#)	Temperature Treatment	pCO_2/pH Treatment	pH_T (at 25 °C)
01 June	HT1	16 °C	Ambient	7,683
				7,701
				7,729
	HT2		OA	7,633
				7,635
				7,629
	HT1	20 °C	Ambient	7,729
				7,737
				7,754
	HT2		OA	7,73
				7,735
				7,734
	HT1	12 °C	Ambient	7,796
				7,791
				7,789
	HT2		OA	7,784
				7,778
				7,785
	1 (1)	16 °C	OA	7,501
	1 (2)			7,475
	2 (1)			7,485
	2 (2)			7,484
	3 (1)			7,482
	3 (2)			7,469
	4 (1)			7,508
	4 (2)			7,512
	5 (1)			7,464
	5 (2)			7,454
	6 (1)			7,479
	6 (2)			7,455
	7 (1)		Ambient	7,756
	7 (2)			7,766
	8 (1)			7,769
	8 (2)			7,776

	9 (1)			7,779
	9 (2)			7,778
	10 (1)			7,779
	10 (2)			7,777
	11 (1)			7,775
	11 (2)			7,771
	12 (1)			7,811
	12 (2)			7,819
	13 (1)	20 °C	OA	7,506
	13 (2)			7,52
	14 (1)			7,52
	14 (2)			7,514
	15 (1)			7,527
	15 (2)			7,536
	16 (1)			7,535
	16 (2)			7,536
	17 (1)			7,53
	17 (2)			7,531
	18 (1)			7,558
	18 (2)			7,538
	19 (1)		Ambient	7,944
	19 (2)			7,957
	20 (1)			7,912
	20 (2)			7,909
	21 (1)			7,915
	21 (2)			7,92
	22 (1)			7,906
	22 (2)			7,903
	23 (1)			7,937
	23 (2)			7,939
	24 (1)			7,946
	24 (2)			7,949
	25 (1)	12 °C	OA	7,448
	25 (2)			7,443
	26 (1)			7,485
	26 (2)			7,457
	27 (1)			7,453
	27 (2)			7,452
	28 (1)			7,496
	28 (2)			7,493
	29 (1)			7,445

	29 (2)			7,443
	30 (1)			7,434
	30 (2)			7,435
	31 (1)		Ambient	7,816
	31 (2)			7,843
	32 (1)			7,834
	32 (2)			7,838
	33 (1)			7,833
	33 (2)			7,832
	34 (1)			7,832
	34 (2)			7,836
	35 (1)			7,834
	35 (2)			7,835
	36 (1)			7,838
	36 (2)			7,846
07 June	HT1	16 °C	Ambient	7,691
				7,695
				7,701
	HT2		OA	7,556
				7,557
				7,559
	HT1	20 °C	Ambient	7,651
				7,657
				7,661
	HT2		OA	7,697
				7,689
				7,695
	HT1	12 °C	Ambient	7,724
				7,723
				7,726
	HT2		OA	7,743
				7,74
				7,745
	2 (1)	16 °C	OA	7,474
	2 (2)			7,473
	3 (1)			7,463
	3 (2)			7,46
	9 (1)		Ambient	7,746
	9 (2)			7,749
	11 (1)			7,73
	11 (2)			7,728

	15 (1)	20 °C	OA	7,541
	15 (2)			7,542
	17 (1)			7,539
	17 (2)			7,54
	21 (1)		Ambient	7,788
	21 (2)			7,791
	24 (1)			7,778
	24 (2)			7,792
	26 (1)	12 °C	OA	7,541
	26 (2)			7,522
	30 (1)			7,468
	30 (2)			7,459
	32 (1)		Ambient	7,726
	32 (2)			7,728
	36 (1)			7,72
	36 (2)			7,722
12 June	HT1	16 °C	Ambient	7,664
				7,679
				7,687
	HT2		OA	7,539
				7,528
				7,526
	HT1	20 °C	Ambient	7,669
				7,708
				7,703
	HT2		OA	7,722
				7,731
				7,734
	HT1	12 °C	Ambient	7,71
				7,732
				7,738
	HT2		OA	7,738
				7,736
				7,735
	1 (1)	16 °C	OA	7,439
	1 (2)			7,423
	5 (1)			7,449
	5 (2)			7,435
	7 (1)		Ambient	7,832
	7 (2)			7,844
	10 (1)			7,834

	10 (2)			7,83
	13 (1)	20 °C	OA	7,509
	13 (2)			7,534
	18 (1)			7,512
	18 (2)			7,508
	20 (1)		Ambient	7,946
	20 (2)			7,949
	23 (1)			7,887
	23 (2)			7,884
	27 (1)	12 °C	OA	7,428
	27 (2)			7,425
	29 (1)			7,435
	29 (2)			7,425
	31 (1)		Ambient	7,795
	31 (2)			7,795
	35 (1)			7,795
	35 (2)			7,788
15 June	HT1	16 °C	Ambient	7,665
				7,68
				7,688
	HT2		OA	7,418
				7,407
				7,406
	HT1	20 °C	Ambient	7,725
				7,734
				7,741
	HT2		OA	7,764
				7,76
				7,756
	HT1	12 °C	Ambient	7,726
				7,73
				7,737
	HT2		OA	7,728
				7,73
				7,726
	3 (1)	16 °C	OA	7,47
	3 (2)			7,459
	5 (1)			7,46
	5 (2)			7,438
	8 (1)		Ambient	7,806
	8 (2)			7,818

	11 (1)			7,833
	11 (2)			7,835
	13 (1)	20 °C	OA	7,501
	13 (2)			7,523
	18 (1)			7,515
	18 (2)			7,51
	20 (1)		Ambient	7,864
	20 (2)			7,864
	23 (1)			7,864
	23 (2)			7,867
	26 (1)	12 °C	OA	7,418
	26 (2)			7,416
	29 (1)			7,408
	29 (2)			7,417
	32 (1)		Ambient	7,797
	32 (2)			7,796
	35 (1)			7,79
	35 (2)			7,792
12 July	1 (1)	16 °C	OA	7,478
	1 (2)			7,464
	2 (1)			7,438
	2 (2)			7,487
	3 (1)			7,475
	3 (2)			7,463
	4 (1)			7,449
	4 (2)			7,479
	5 (1)			7,466
	5 (2)			7,466
	6 (1)			7,48
	6 (2)			7,495
	7 (1)		Ambient	7,752
	7 (2)			7,766
	8 (1)			7,767
	8 (2)			7,763
	9 (1)			7,745
	9 (2)			7,76
	10 (1)			7,786
	10 (2)			7,807
	11 (1)			7,796
	11 (2)			7,797
	12 (1)			7,792

	12 (2)			7,792
	13 (1)	20 °C	OA	7,56
	13 (2)			7,534
	14 (1)			7,563
	14 (2)			7,557
	15 (1)			7,573
	15 (2)			7,572
	16 (1)			7,554
	16 (2)			7,557
	17 (1)			7,553
	17 (2)			7,559
	18 (1)			7,548
	18 (2)			7,542
	19 (1)		Ambient	7,884
	19 (2)			7,87
	20 (1)			7,891
	20 (2)			7,898
	21 (1)			7,878
	21 (2)			7,869
	22 (1)			7,877
	22 (2)			7,872
	23 (1)			7,869
	23 (2)			7,874
	24 (1)			7,885
	24 (2)			7,883
	25 (1)	12 °C	OA	7,473
	25 (2)			7,486
	26 (1)			7,526
	26 (2)			7,527
	27 (1)			7,459
	27 (2)			7,454
	28 (1)			7,467
	28 (2)			7,471
	29 (1)			7,464
	29 (2)			7,462
	30 (1)			7,471
	30 (2)			7,472
	31 (1)		Ambient	7,802
	31 (2)			7,804
	32 (1)			7,802
	32 (2)			7,801

	33 (1)			7,779
	33 (2)			7,797
	34 (1)			7,782
	34 (2)			7,789
	35 (1)			7,808
	35 (2)			7,806
	36 (1)			7,803
	36 (2)			7,802
19 July	1 (1)	16 °C	OA	7,51
	1 (2)			7,506
	6 (1)			7,488
	6 (2)			7,474
	8 (1)		Ambient	7,825
	8 (2)			7,842
	11 (1)			7,843
	11 (2)			7,836
	15 (1)	20 °C	OA	7,558
	15 (2)			7,544
	17 (1)			7,527
	17 (2)			7,528
	21 (1)		Ambient	7,909
	21 (2)			7,914
	23 (1)			7,848
	23 (2)			7,865
	25 (1)	12 °C	OA	7,469
	25 (2)			7,433
	29 (1)			7,428
	29 (2)			7,436
	31 (1)		Ambient	7,786
	31 (2)			7,788
	36 (1)			7,768
	36 (2)			7,777

Supplementary Table 3: Results from the analysis of variance (*anova*) for linear models (Gaussian family), with temperature (12°, 16°, 20 °C), pCO_2 concentrations (ambient [400µatm] and OA [1200µatm]), locations (Las Docas and El Tabo) and experimental trials (I-II) as fixed effects. Only results of the best models are reported, after selection of the lowest AICc score. Significant p-values in bold font. ω^2 represents magnitude of effects values within each response variable (e.g., growth rate), and % represents percentage of ω^2.

	Df	Sum Sq	Mean Sq	F value	P value	ω^2	%
**Growth rate**							
pCO_2	1	4.426	4.426	0.519	0.474	-0.004	-0.4
Temperature	2	345.595	172.798	20.282	**0.000**	0.342	34.2
Location	1	54.72	54.72	6.423	**0.014**	0.048	4.8
Trial	1	0.788	0.788	0.092	0.762	-0.008	-0.8
Residuals	64	545.259	8.52	-	-	-	-
**Photosynthetic rate**							
pCO_2	1	22.279	22.279	1.914	0.171	0.012	1.2
Temperature	2	20.845	10.422	0.895	0.413	-0.003	-0.3
Location	1	23.223	23.223	1.995	0.163	0.013	1.3
Trial	1	25.627	25.627	2.202	0.143	0.016	1.6
Residuals	66	768.278	11.641	-	-	-	-
**Alfa (α)**							
pCO_2	1	0.002	0.002	0.201	0.656	-0.011	-1.1
Temperature	2	0.016	0.008	1.05	0.356	0.001	0.1
Location	1	0.012	0.012	1.543	0.219	0.008	0.8
Trial	1	0.013	0.013	1.682	0.199	0.01	1
Residuals	64	0.486	0.008	-	-	-	-
**ETRmax**							
pCO_2	1	0.001	0.001	0.035	0.853	-0.013	-1.3
Temperature	2	0.143	0.072	3.044	0.055	0.054	5.4
Location	1	0.001	0.001	0.039	0.844	-0.013	-1.3
Trial	1	0.111	0.111	4.727	**0.033**	0.049	4.9
Residuals	64	1.506	0.024	-	-	-	-
**Ek**							
pCO_2	1	5.796	5.796	0	0.989	-0.015	-1.5
Temperature	2	53166.79	26583.395	0.885	0.418	-0.003	-0.3
Location	1	23425.6	23425.6	0.78	0.381	-0.003	-0.3
Trial	1	19973.688	19973.688	0.665	0.418	-0.005	-0.5
Residuals	64	1923312.246	30051.754	-	-	-	-
**Fv/Fm**							
pCO_2	1	0.001	0.001	0.338	0.563	-0.008	-0.8
Temperature	2	0.02	0.01	3.681	**0.031**	0.067	6.7
Location	1	0.009	0.009	3.262	0.076	0.028	2.8
Trial	1	0.011	0.011	4.096	**0.047**	0.039	3.9
Residuals	64	0.173	0.003	-	-	-	-
**NR activity**							

pCO_2	1	0.2551	0.2551	3.2442	0.076	0.023	2.3
Temperature	2	0.4028	0.2014	2.5608	0.085	0.032	3.2
Location	1	0.5549	0.5549	7.0559	**0.01**	0.0621	6.21
Trial	1	1.3428	1.3428	17.075	**0.0001**	0.1649	16.49
Residuals	64	5.0328	0.0786	-	-	-	-
**CA activity**							
pCO_2	1	2.2	2.2	1.309	0.257	0.004	0.4
Temperature	2	24.535	12.267	7.301	**0.001**	0.148	14.8
Location	1	12.793	12.793	7.614	**0.008**	0.077	7.7
Trial	1	3.172	3.172	1.888	0.175	0.01	1
Residuals	59	99.135	1.68	-	-	-	-
**Chl a**							
pCO_2	1	0	0	0.0001	0.994	-0.0077	-0.77
Temperature	2	0.3965	0.1983	15.278	**0.000**	0.2186	21.86
Location	1	0.0097	0.0097	0.7437	0.392	-0.002	-0.2
Trial	1	0.0003	0.0003	0.022	0.883	-0.0075	-0.75
Temp×Location	2	0.0326	0.0163	1.2553	0.293	0.0039	0.39
Temp×Trial	2	0.3549	0.1775	13.674	**0.000**	0.1941	19.41
Location×Trial	1	0.1001	0.1001	7.7117	**0.007**	0.0514	5.14
Temp×Location×Trial	2	0.0614	0.0307	2.3662	0.103	0.0209	2.09
Residuals	56	0.7268	0.013	-	-	-	-
**Chl c**							
pCO_2	1	0	0	0.005	0.943	-0.008	-0.8
Temperature	2	0.081	0.04	3.07	0.054	0.034	3.4
Location	1	0.143	0.143	10.865	**0.002**	0.081	8.1
Trial	1	0.161	0.161	12.237	**0.001**	0.092	9.2
Temp×Location	2	0.046	0.023	1.738	0.185	0.012	1.2
Temp×Trial	2	0.152	0.076	5.808	**0.005**	0.079	7.9
Location×Trial	1	0.096	0.096	7.347	**0.009**	0.052	5.2
Temp×Location×Trial	2	0.175	0.087	6.66	**0.003**	0.093	9.3
Residuals	56	0.735	0.013	-	-	-	-
**Fucoxanthin**							
pCO_2	1	0.013	0.013	1.35	0.25	0.003	0.3
Temperature	2	0.153	0.076	7.677	**0.001**	0.119	11.9
Location	1	0.016	0.016	1.622	0.208	0.006	0.6
Trial	1	0.043	0.043	4.359	**0.041**	0.03	3.0
Temp×Location	2	0.072	0.036	3.616	**0.033**	0.047	4.7
Temp×Trial	2	0.07	0.035	3.543	**0.036**	0.045	4.5
Location×Trial	1	0.085	0.085	8.534	**0.005**	0.067	6.7
Temp×Location×Trial	2	0.097	0.048	4.858	**0.011**	0.069	6.9
Residuals	56	0.557	0.01	-	-	-	-
**Tissue N content**							
pCO_2	1	0.019	0.019	0.635	0.428	-0.002	-0.2
Temperature	2	0.541	0.27	9.026	**0.000**	0.079	7.9
Location	1	0.146	0.146	4.871	**0.031**	0.019	1.9
Trial	1	3.397	3.397	113.45	**0.000**	0.551	55.1
Residuals	66	1.976	0.03	-	-	-	-

**Tissue C content**							
pCO_2	1	3.4137	3.4137	1.4943	0.226	0.0015	0.15
Temperature	1	470.8639	470.8639	206.12	**0.000**	0.6376	63.76
Location	2	36.9065	18.4532	8.0779	**0.0008**	0.044	4.4
Trial	1	42.0651	42.0651	18.414	**0.0001**	0.0541	5.41
pCO_2×Temp	2	10.0441	5.0221	2.1984	0.12	0.0075	0.75
pCO_2×Location	1	2.9389	2.9389	1.2865	0.261	0.0009	0.09
Temp×Location	2	3.7009	1.8504	0.81	0.45	-0.0012	-0.12
pCO_2×Temp×Location	2	27.9267	13.9634	6.1124	**0.004**	0.0318	3.18
Residuals	59	134.7805	2.2844	-	-	-	-
**CN ratio**							
pCO_2	1	0.0018	0.0018	0.004	0.95	-0.0065	-0.65
Temperature	2	39.7489	19.8745	42.715	**0.000**	0.5432	54.32
Location	1	0.5293	0.5293	1.1375	0.29	0.0009	0.09
Trial	1	0.0115	0.0115	0.0248	0.875	-0.0063	-0.63
Residuals	66	30.7084	0.4653	-	-	-	-
**NR gene**							
pCO_2	1	0.288	0,288	8.924	**0.008**	0.037	3.7
Temperature	2	3.815	1.908	59.108	**0.000**	0.537	53.7
Location	1	0.271	0.271	8.392	**0.010**	0.034	3.4
pCO_2×Temp	2	0.711	0.355	11.010	**0.001**	0.093	9.3
pCO_2×Location	1	0.001	0.001	0.043	0.838	-0.004	0.4
Temp×Location	2	1.307	0.654	20.255	**0.000**	0.178	17.8
pCO_2×Temp×Location	2	0.005	0.002	0.072	0.931	-0.009	0.9
Residuals	17	0.549	0.032	-	-	-	-
**CA gene**							
pCO_2	1	0.010	0.010	0.070	0.795	-0.006	0.60
Temperature	2	6.464	3.232	22.006	**0.000**	0.291	29.0
Location	1	6.589	6.589	44.859	**0.000**	0.303	30.3
pCO_2×Temp	2	0.051	0.025	0.173	0.843	-0.011	1.10
pCO_2×Location	1	1.092	1.092	7.436	**0.014**	0.045	4.50
Temp×Location	2	2.771	1.386	9.433	**0.002**	0.117	11.7
pCO_2×Temp×Location	2	1.605	0.803	5.464	**0.015**	0.062	6.20
Residuals	17	2.497	0.147	-	-	-	-
**SP gene**							
pCO_2	1	0.2693	0.2693	0.4737	0.5000	-0.0059	-0.59
Temperature	2	156.292	78.146	137.446	**0.0002**	0.2891	28.91
Location	1	0.1455	0.1455	0.2559	0.6190	-0.0084	-0.84
pCO_2×Temp	2	0.3425	0.1712	0.3012	0.7435	-0.0158	1.58
pCO_2×Location	1	21.968	21.968	38.638	0.065	0.0324	3.24
Temp×Location	2	200.773	100.386	176.563	**0.000**	0.3779	37.79
pCO_2×Temp×Location	2	0.6494	0.3247	0.5711	0.5748	-0.0097	-0.97
Residuals	18	102.340	0.5685	-	-	-	-
